# Transformation-oriented leadership in German hospitals: beneficial attributes and competencies

**DOI:** 10.1093/eurpub/ckac131.379

**Published:** 2022-10-25

**Authors:** J Kleine, J Köppen, C Maier

**Affiliations:** 1 Department of Health Care Management, Technische Universität Berlin, Berlin, Germany; 2 European Observatory on Health Systems and Policies, Brussels, Belgium

## Abstract

**Introduction:**

Due to a shortage of health professionals and economic pressure, many German hospitals are required to transform the clinical work environment to increase job satisfaction, but also to attract and retain health professionals. Leadership is a key factor for the successful implementation of organization-wide change. The aim of this study was to identify the attributes and competencies among leaders that are beneficial for implementing and managing hospital-wide transformations.

**Methods:**

A qualitative study design. In 2020, 18 face-to-face, semi-structured interviews were conducted with chief nursing officers, ward managers, nurses and physicians in five German hospitals which have started implementing a hospital-wide transformation (e.g. Magnet® or Pathway®). Interviews were recorded and transcribed verbatim. Data were analyzed in Atlas.ti using the content analysis method according to Mayring.

**Results:**

Results show five beneficial leadership elements to instigate and steer hospital-wide transformation: (1) Charismatic leaders are role models and idealists with well-communicated visions that are grounded in clinical practice and reflect the clinical practitioners. (2) Mentally strong leaders have courage, stamina, and are resilient. (3) Empowering leaders are highly supportive and increase the intrinsic motivation of employees. (4) Imparting interprofessional appreciation refers to leaders who cultivate a respectful relationship with persons from other professional groups and recognize their daily performance. (5) Agile leaders are well and quickly accessible for employees and respond situationally to changing demands in everyday work.

**Conclusions:**

Interviewees described characteristics of hospital leaders as success factors for establishing and maintaining continuous change processes. Charismatic and supportive leaders are critical to transform the hospitals’ culture and values. In addition, equitable interprofessional collaboration is of utmost importance.

**Key messages:**

• Charismatic, agile, empowering leaders can have a positive impact on system-wide change processes.

• The successful transformation of hospitals’ work environment needs interprofessional appreciation and the mutual acknowledgement of competences.

